# Opportunities to design better computer vison-assisted food diaries to support individuals and experts in dietary assessment: An observation and interview study with nutrition experts

**DOI:** 10.1371/journal.pdig.0000665

**Published:** 2024-11-27

**Authors:** Chia-Fang Chung, Pei-Ni Chiang, Connie Ann Tan, Chien-Chun Wu, Haley Schmidt, Aric Kotarski, David Guise

**Affiliations:** 1 Computational Media, Baskin School of Engineering, University of California Santa Cruz, Santa Cruz, California, United States of America; 2 Luddy School of Informatics, Computing, and Engineering, Indiana University Bloomington, Bloomington, Indiana, United States of America; 3 Department of OB/GYN, Indiana University School of Medicine, Indianapolis, Indiana, United States of America; 4 Department of Biostatistics and Health Data Science, Indiana University School of Medicine, Indianapolis, Indiana, United States of America; University of Waterloo, CANADA

## Abstract

Automatic visual recognition for photo-based food diaries is increasingly prevalent. However, existing tools in food recognition often focus on food classification and calorie counting, which may not be sufficient to support the variety of food and healthy eating goals people have. To understand how to better design computer-vision-based food diaries to support healthy eating, we began to examine how nutrition experts, such as dietitians, use the visual features of food photos to evaluate diet quality. We conducted an observation and interview study with 18 dietitians, during which we asked the dietitians to review a seven-day photo-based food diary and fill out an evaluation form about their observations, recommendations, and questions. We then conducted follow-up interviews to understand their strategies, needs, and challenges of photo diary review. Our findings show that dietitians used the photo features to understand long-term eating patterns, diet variety, eating contexts, and food portions. Dietitians also adopted various strategies to achieve these understandings, such as grouping photos to find patterns, using color to estimate food variety, and identifying background objects to infer eating contexts. These findings suggest design opportunities for future compute-vision-based food diaries to account for dietary patterns over time, incorporate contextual information in dietary analysis, and support collaborations between nutrition experts, clients, and computer vision systems in dietary review and provide individualized recommendations.

## Author summary

Many smartphone-based food journaling apps increasingly use computer vision technology to recognize food content, calculate calories, and identify nutrients from food photos. While these applications have the potential to provide healthy eating recommendations based on the results of food image recognition, existing systems often do not provide satisfying results. In this study, we turn to human nutrition experts–dietitians–to understand how to better design computer vision-assisted food journals to support healthy eating assessment and review. Our goal was to learn the types of photo features that human experts use and how they use those features in dietary assessment. These strategies will then support researchers and designers to create computational processes and features in digital food journaling technology. Using interviews and observation, we found that dietitians did not focus on calorie or nutrient estimation from a single photo when reviewing food photos. Instead, they looked across multiple photos and adopted various visual strategies to understand eating patterns, variety, contexts, and portions. These results provide various directions for future digital photo-based food diaries and computer vision technology to support healthy eating.

## Introduction

### Overview

Supporting healthy eating is important but challenging. Many people track what they eat and work with trained experts, such as dietitians, to understand their diet intake, manage chronic conditions, and find opportunities to improve their eating behavior [1–3]. Traditional dietary assessment methods include 24-hour dietary recalls, food frequency questionnaires, and text-based food diaries [4]. These methods play a crucial role in supporting experts to understand individual food intake and provide personalized recommendations. However, both 24-hour recalls and food frequency questionnaires rely on individual memory and could be prone to error [4]. While text-based food diaries could be more based on “fact,” they are burdensome for people to use [5,6], create negative nudges to eating [6], and can be challenging for providers to review in practice [7].

### Photo-based dietary assessment

In recent years, photo-based diaries have become prevalent. With smartphones and wearable cameras, taking a photo of food is easier and more socially acceptable than writing down a list of ingredient items [3,6,8]. The act of taking photos also helps people be more mindful of what they eat and prompts reflection [6,8]. Image-based dietary assessment (IBDA) has also increasingly been adopted as a formal dietary assessment method, with a 2022 review showing that 49% of research on smartphone-based dietary assessment tools use photo-based assessment[9]. IBDA allows dietitians to better estimate portion sizes and assess macronutrients, compared to recalls or food frequency questionnaires [10,11]. Dietitians can have a more accurate understanding of the actual consumption if they can see the before and after pictures [10]. Research also shows that image-based diaries have greater reproducibility than text-based diaries [12] and reduce participant underreporting rates by 6 to 8% [13]. These diaries could also potentially include a broader set of users to whom traditional text-based diaries or recall methods are challenging [14,15]. When reviewing photos with patients or clients, food photos provide visual examples to help diabetes educators communicate with patients [3]. Contextual information captured in food photos also supported individuals to work with health experts to identify triggers or behavior change opportunities [16].

While photo-based diaries potentially decrease the burden of collecting food data, reviewing and analyzing a large number of photos can be challenging [9,10]. Prior research has attempted to provide dietitian support by designing visual summaries [16] or data visualizations [17,18]. Some systems also allow individuals to supplement dietary information with voice input or environmental information to support data analysis (e.g., [19,20]). Some studies included detailed instructions, such as only taking photos from specific angles or including a color reference (e.g., [21,22]). Similarly, not everyone has consistent access to nutrition experts or resources for dietary assessment and recommendation. Many research and commercial systems have started to adopt an automatic analysis approach for photo-based dietary assessment ([9,10,15]).

### Automatic food photo analysis in dietary assessment

To support on-demand, personalized reviews, many food journaling apps (e.g., Bitesnap [23] and Calorie Mama [24]) adopt computer vision algorithms that analyze food content from photos. However, despite the widespread, individual uptake of photo-based diaries, there are still limited successes in how computer vision could support individuals or dietitians in reviewing diet quality [25,26]. Research in the automatic recognition of food images is prevalent, but the results have been inconsistent due to challenges such as food segmentation, volume estimation, and image quality [25,27,28]. This line of research predominately uses food photos as a benchmark in image segmentation or classification, focusing on categorizing foods into mutually exclusive categories of objects (e.g., chicken salad or alfredo pasta) [29–34]. However, these pre-defined categories may not align with the variety of food people eat every day. Many existing algorithms are also often trained on idealized food images collected from the web (e.g., [31,35,36]) or are focused on restaurant food (e.g., [30]). As a result, researchers have found these techniques and apps work poorly on the food photos that real people take in their daily lives [25,26]. Research examining how dietitians work with automatic image analysis is still in the preliminary stage, with existing studies suggesting the need to allow dietitians further customization or include dietitian knowledge in automatic analysis [37,38]. As dietary guidelines gradually expand from calorie restriction to diet quality improvement (e.g., focusing on healthy eating patterns, variety, and nutrient density [39,40]), existing systems focusing on caloric or nutrient estimation (e.g., [29,41]) also are not sufficient to provide appropriate assessment and recommendation.

### Research goal

To understand how computer vision and automatic image analysis could support dietary assessment, this research sets out to examine how dietitians, human experts in dietary assessment, review food photos, what they focus on when reviewing these diaries, and how systems could support them and their clients in reviewing photo-based diaries. Adding to the effort of translating dietitian diagnosis process into computational inference knowledge [42], our research seeks to examine dietitian goals, processes, and challenges of reviewing photo-based diaries to support healthy eating goals. A better understanding of dietitian review process using photo-based diaries could better support future system design in personalized dietary assessment and consultation.

To achieve this goal, we conducted an observation and interview study with 18 participants, including registered dietitians and dietitian interns, to understand their goals and processes while reviewing photo diaries. In addition to the observation and interviews, we also asked participants to write down their observations from the photos, questions they had while reviewing food photos, and dietary recommendations to the clients who took the food photos. Our study contributes to the digital health community:

- An understanding of dietitian review process, strategies, and goals of using photo-based food diaries to support healthy eating.
- Research and design opportunities for computer vision technology to support individuals and dietitians in photo-based dietary data collection, integration, and reflection to support healthy eating.

## Methods

We conducted an observation and interview study with 18 participants, who are registered dietitians and dietitian interns in the United States. We recruited participants through mailing lists associated with a research university and word of mouth. The study protocol was approved by the university Institutional Review Board (IRB).

### Observation and interview procedures

**Fig 1:**
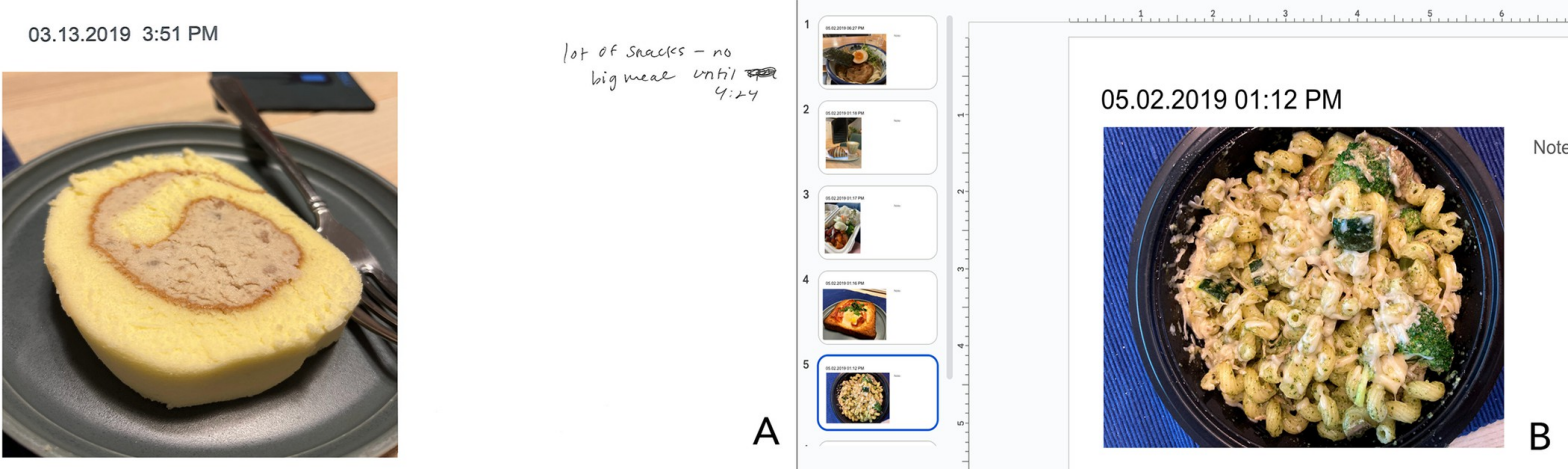
Seven-day photo-based food diaries for dietitian participants review. During in-person observations, we printed out each slide to allow participants to shuffle, annotate, and take notes (A). During the remote interview, we used Google Slides to share the photos to allow participants to type their notes and rearrange slides online (B).

**Fig 2:**
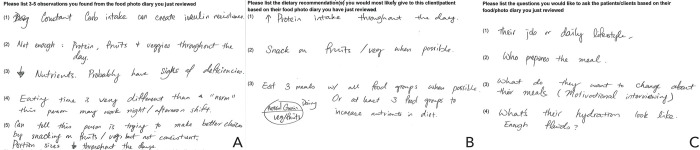
Participants filled out evaluation forms at the end of the food diary review. These forms asked about participant observation from the diary (A), recommendations derived from the review (B), and questions they had for the clients (C).

In the observation session, we provided each participant with a 7-day photo-based food diary using the dataset from a prior IRB-approved study. Each diary contains 30–45 (M = 37.29, SD = 5.29) food photos. Participants in this prior study were healthy with various types of general healthy eating goals, ranging from managing weight, balancing diet, to eating more vegetables or less sugar. These participants were asked to take photos of their food for a week, including both weekdays and weekends. They received a set of instructions for food photo-taking. These instructions include guidelines to ensure the quality of the photos, such as making sure all food can be seen in the photo or including containers or utensils when possible for portion reference, and those to ensure the privacy of others, such as not taking photos in facilities restricted in photo taking or obtaining agreement in private space. These participants took 4–7 (M = 5.8, SD = 1.8) photos per day and 30–72 (M = 40.9, SD = 11.9) photos over the seven-day period. All participants from this prior study consented for the research team to use their diaries for future studies. In this current study, we provided each dietitian with a different seven-day diary because our goal was to understand the variety of ways dietitians make use of photo-based diaries, instead of comparing the dietary analysis results across participants. We printed out each food photo with the date/time when the photo was taken on letter-size paper. We left half of the paper blank for dietitians to annotate as they wish (Fig 1A). We chose to print the photos on paper to allow participants to interact with the photos (e.g., highlight, annotate, change the sequence, group) without the constraints of digital devices (e.g., screen size). We print all the photos using the same printer with the same color profile to ensure all participants have the same color and image quality experience with the food photos. We also provided the diary keeper’s health goals and basic demographic information (e.g., gender, age, and BMI) to the dietitian participants. These review sessions are similar to dietary consultation sessions without patients or clients present, such as when a dietitian works on patient diaries before or between visits. Dietitian participants were allowed to spend as much or as little time as they wished for the review. We asked participants to think aloud when they reviewed the photos. We also asked them to fill out an evaluation form at the end of the review to provide additional information about the photo-based diary review: (1) Open-ended observations they found from the diary, (2) Open-ended dietary recommendations they would most likely give to this client, and (3) Questions they would like to ask the client based on the food photos they reviewed (Fig 2).

The observations lasted from 30 to 40 minutes. After finishing reviewing the photos, we conducted a 30-minute semi-structured interview to further probe participant review experiences and get more detailed explanations for the review process participants went through to develop assessments and recommendations. We first asked about participant experience with dietary assessment and with photo-based diary review. We then asked participants to go through their review process during the observation study. Finally, we used the observation notes to follow up on why and how they focused on specific aspects and information in the photo-based diaries. Each participant was compensated with a $20 Amazon gift card for reviewing a seven-day photo-based food diary and a $20 Amazon gift card for the interview session.

We conducted the first nine studies in person. However, as the COVID-19 pandemic progressed, we moved to remote observations and interviews using video conferencing tools, such as Zoom [43], Google Meet [44], and FaceTime [45] with the last nine participants. To support remote observations, we used Google Slides [46] to present photo diaries to each participant, with each slide showing one photo and its timestamp (Fig 1B). While we strove to present the data similar to the in-person studies, subtle differences in photo presentation, such as the color schemes in participants devices and paper printouts, could result in participants seeing photos differently. We chose Google Slides because it provides general interaction features (e.g., annotation, comments, grouping, rearrangement). We also asked participants to fill out the evaluation forms by typing on the last three slides. During the review session, participants were allowed to change the order of the photos and switch from slide view to grid view to support their review process.

### Participants

Among these 18 participants, there were ten outpatient dietitians, two inpatient dietitians, five private practitioners, and one dietitian with multiple roles. All participants were female, with dietary assessment experience ranging from 0.5 to 35 years. Eight participants had experience reviewing photo-based food diaries. We strove to recruit participants with varied dietary assessments and photo-based food diary review experiences to explore a diverse set of strategies nutrition experts might use. Detailed information about participant demographics can be found in Table 1.

### Data analysis techniques

All interviews and observations were audio-recorded and transcribed for further analysis. We analyzed the data in three phases, using a mix of inductive and deductive methods. We first conducted an affinity diagram analysis [47] of the data from the first six participants to understand the common foci and issues dietitians had while reviewing photo-based food diaries. Four researchers transformed these six interview transcripts into approximately 300 affinity notes, with each note representing a distinct point or idea extracted from the interview transcripts. The same researchers then iteratively organized these notes into 25 categories. The whole research team then verified and discussed these emergent categories with the whole research team. We then used these themes to create an initial codebook. Based on the codebook, we iteratively coded the data from another six participants to form a deeper understanding of dietitian practices in reviewing food photos. Four researchers independently coded the same transcript and met to resolve ambiguities in the codebook. The whole research team also met weekly to discuss and iterate on the coding decisions. After coding these six transcripts, we used these codes to analyze the rest of the data. Finally, the research team re-reviewed all transcripts to reflect the whole codebook. Through this process, we identified an additional seven themes, resulting in a total of 32 themes focusing on dietitian strategies and information sought from photo diaries. During the analysis, we also referenced the evaluation forms to support the understanding of these strategies and the information dietitians looked for to support their review goals. We shared the resulting themes with two dietitians in member-checking sessions to confirm key insights representing participant goals, processes, and challenges.

**Table 1:** Participant demographic information.

Participants	Occupation	Years of Professional Practices	Experience in reviewing photos-based food diaries	In-person or remote observation
P01	Outpatient dietitian	2	Yes	In-person
P02	Outpatient dietitian	27	Yes	In-person
P03	Inpatient dietitian intern	0.5	No	In-person
P04	Inpatient dietitian intern	0.5	No	In-person
P05	Outpatient dietitian	11	No	In-person
P06	Outpatient dietitian	3	Yes	In-person
P07	Private practitioner	5	Yes	In-person
P08	Private practitioner	15	No	In-person
P09	Outpatient dietitian	1.5	No	In-person
P10	Outpatient dietitian	30	Yes	Remote
P11	Private practitioner	35	No	Remote
P12	In- & out-patient dietitian	15	No	Remote
P13	Private practitioner	8	Yes	Remote
P14	Outpatient dietitian	14	Yes	Remote
P15	Outpatient dietitian	11	No	Remote
P16	Private practitioner	1	Yes	Remote
P17	Outpatient dietitian	13	No	Remote
P18	Outpatient dietitian	10	No	Remote

## Results

Participants on average spent 34.67 (SD = 5.60) minutes reviewing a seven-day photo diary. In in-person observations, we saw many participants spread the photos on the table and grouped them in different ways to support their review. In remote observations, participants flipped through all the photos quickly and then moved the slides to help them answer questions they had about client diets.

Our findings found that participants focused on understanding eating patterns, variety, and contexts in their review of food photos. They also used visual features, such as color and background artifacts to support these strategies. While not currently available in the photos, dietitians envision using food photos to evaluate the sufficiency of intake in the future. We summarize these strategies and the different ways participants used visual features to support these strategies in Table 2. We did not see major differences in these strategies and the visual features used between in-person and online observations. However, in the online observation sessions, we did provide technical support to facilitate the review when participants indicated the need for specific interaction features but did not know how to use them (e.g., selecting multiple photos at the same time).

**Table 2:** Participant strategies for using visual features in food photo reviews.

Food photo review strategies	How visual features were used	Examples
Understand client eating patterns	Crafting a view that allows viewing all photos together	• In-person: laying out all photos on the table (P04) • Online: switched to “Grid View” to view all photos (P13)
	Counting food item frequency	• Circling high-frequency food (P04) • Checking how often “good” vs. “bad” foods in the photos (P13)
	Grouping photos taken at similar time	• Checking time between meals (P03) • Using timestamp + content to distinguish meal vs. snacks (P01)
Ensure clients have a variety of intake	Use color to determine food group balance	• Photos with less diverse colors indicate a lack of vegetables or fruits (P01, P03)
	Use visual division of a plate to estimate food group distribution	• Estimate how much vegetables, protein, and grains take up in a plate (P04, P12, P17)
Infer how and where clients ate	Use background artifacts to understand eating contexts (e.g., location, social contexts, and food preparation abilities)	• Location: Fancy plates are more likely to be in a restaurant setting (P13) • Social settings: Various types of food on the same plate and disposable utensils infer potential party setting (P15) • Food preparation: Kitchenware or ingredient packages show possibilities of making food at home (P09, P16)
Sufficiency of client intake	Suggesting additional photo-taking strategies to improve understanding	• Taking before-and-after photos to understand actual portions consumed (P04, P11) • Including reference objects (P03, P05) • Taking photos from different angles to identify container depth (P13, P16)

### Understand client eating patterns

United States Department of Agriculture (USDA) defines dietary patterns as “*Over the course of any given day*, *week*, *or year*, *individuals consume foods and beverages in combination—a dietary pattern*. *A dietary pattern represents the totality of what individuals habitually eat and drink*, *and the parts of the pattern act synergistically to affect health*” [39]. Most participants started the review by looking for meal patterns across the whole set (seven days) of photos. They wanted to understand the patterns of common food these clients consumed, the time of date they ate, and the differences between their weekday and weekend food choices. While these goals may be similar to what dietitians would do with other types of dietary assessment tools (e.g., text-based diaries), participants actively used the visuals from the photos to support identifying these patterns.

Participants commented on how photos made it easy for them to look at the overall eating behavior, instead of focusing on individual meal entries. For example, when reviewing the diary using Google Slides, P13 switched the presentation mode to “Grid View,” skimmed the photos, and told us how being able to look at photos together gave her an overview of the client eating behaviors and decisions. She mentioned that she would not fixate on individual photos in the analysis but would focus on how the client is eating across the week:

“*I like that with photos it’s easier to look at it as a whole and say*, *okay*, *they got their protein from this*, *they got their fat from this*, *they got carbohydrates or vegetables here*. *Because we can’t look at somebody’s diary and judge each individual food*. *Right*? *If I look at this (clicking at one photo) and I see gummy bears and I think*, *‘Oh*, *maybe that’s not a good one’; (Clicking at another photo) I see ice cream and then I see apple sauce*. *I’m like*, *‘What the heck is this person eating*?*’ But to see the whole picture*, *throughout a week’s time I had a day that maybe I had ice cream and candy and all of this*, *but every other day I limited those things*. *From a nutrient standpoint and habits standpoint*, *being able to see the week at a time*, *seeing multiple pictures at a time*, *was helpful for me to understand how they ate*, *how they are*.” (P13; Private practitioner for 8 years)

Many participants also thought that photos helped them quickly identify what foods clients often eat, which helped them understand client dietary preferences or restrictions. For example, when P04 laid out the photos on the table, she immediately noticed that the breakfast food items, such as eggs and hash browns, appeared frequently in the diary. She circled those foods in the photos, pointed to them one by one, and noted the frequency of these types of food showing up in the seven-day diary. She then made some hypotheses on potential client lifestyle or preferences based on this information:

“*It looks like they like breakfast foods because you can see the eggs (pointing to one photo) and the hash brown (pointing to another photo), like sausage and French toast (pointing to a third photo). So maybe their job provides food wherever they’re working, or maybe they’re a breakfast person so they’re still eating breakfast food at this time of the day.*” (P04; Inpatient dietitian intern for 6 months)

She then described how she would use this information as a foundation to provide feasible recommendations.

“*So this helps because you can see what they like to eat and then maybe ask how they feel when they eat these things and then try to figure out if they’d be willing to eat any alternatives.*” (P04; Inpatient dietitian intern for 6 months)

Because timestamps were automatically captured when food photos were taken, participants found it easy to use the timestamps along with the visual content of the photos to determine eating patterns. P03 sorted the photos by day and then, within each day, she grouped the foods that were eaten at a similar time together. She then wrote down the time between each photo within the group and attempted to make sense of the short period of time between meals. She pointed to these groups and said:

“*My first impression is that they’re eating a lot all at once. And then they’re not eating for very long in the day. It’s only a short period of the day, later in the day, they’re eating. … (Gesturing at a photo group and pointing at two photos taken within 15 minutes apart) To me that means that they probably weren’t getting full from the meal. 15 minutes later, they were still hungry, so they were eating some more. So maybe they weren’t getting enough at the meal. See?*” (P03; Inpatient dietitian intern for 6 months)

She then followed up with the type of questions she would ask to further understand the client eating habits and patterns:

“*I would kind of ask, try to get an idea of why they’re waiting later in the day to eat. Maybe they slept in or maybe they were not hungry? If they get up at 8:00 AM and don’t eat until four and then eat from 4:00 PM to midnight, that’s when I would say, ‘Well, how does your body fuel itself all day? You’ve just slept for eight hours and then didn’t eat until 4:00 PM’. So I think the time of day itself isn’t the question as much as it is or an issue as much as it is. If they are somebody that just likes to eat for a small window, then we need to see some big changes in that time. If they do have more time to eat and they’re open to it, then I think we need to see that and evaluate that as well.*” (P03; Inpatient dietary intern for 6 months)

Some participants used the visual content as well as the time of the day the food was eaten to distinguish meals and snacks. P01 sorted the photos by day and then grouped them into meals and snacks. She considered that looking at the meal and snack distribution across a day gave her some ideas about how feasible specific diet recommendations would be. For example, client meal choices might be restricted by their work hours and therefore may not be feasible to change.

“*I would look at a day instead of just looking at individual meals, cause if you’re telling a person to change their meal that may be harder. Maybe they won’t have time during work to change their meals. Maybe that snack is the easiest one to change. So we look at the whole day instead of focusing on one meal.”* (P01; outpatient dietitian for 2 years)

P04 used a similar method–by inspecting the visual content and time of day–to separate meals and snacks. She also looked at these groups both across a day and a seven-day period.

"*Majority of them I would group into meals or if I found more snacks, I would put those together like these over here (Pointing to a list of photos on another side of the table). Like their fruit and then this [cake] (pointing at a photo) is like snacks. Then you can kind of come to conclusions when they’re eating, like what times of day, what they’re eating then. And if they’re snacking based on the time of the day there.*" (P04; Inpatient dietary intern for 6 months)

Our findings show that dietitian participants used different strategies to group and review food photos to identify individual patterns. They also tried to determine individual dietary preferences and constraints from these patterns to help them tailor recommendations.

### Ensure clients have a variety of intake

Once participants had an overview of the client eating patterns, their most common review goal was to determine whether the client had a balanced diet or ate enough variety of food. Instead of identifying individual ingredients in each food photo, participants in our study looked at the variety of foods across photos. They used high-level features of the photos, such as colors, to provide overall assessments of the food variety and balance.

P1 showed us how she used color to determine whether participants had the proper balance of food groups: " *(Pointing at several photos one-by-one) There’s not a lot of colors in these food photos*. *So not a lot of fruits and vegetables*" (P01; Outpatient dietitian for 2 years). She went on to explain that the diary showed that the client mainly consumed carbohydrates without having protein, vegetables, and fruits in her meals: “*That could be a problem for most people*, *cause if you’re not eating enough protein or fat or fiber*, *then you’re constantly hungry*." (P01; Outpatient dietitian for 2 years)

Similarly, P03 looked at the color distribution across the whole set of photos and used that to determine the balance of food. Similar to P01, she saw homogeneous color in the photo set and considered the client not eating enough vegetables. “*With this person*, *I could see that he or she is constantly eating a lot of carbohydrates*. *There are a lot of the same kinds of foods*, *not a lot of vegetables*.*”* (P03; Inpatient dietary intern for 6 months)

When looking closely into individual photos, participants thought having client food photos made it easy for them to assess client food composition and make recommendations that clients could follow. Many participants used strategies similar to MyPlate to assess the balance of food. MyPlate is a nutrition guide recommended based on USDA’s Dietary Guidelines for Americans [39]. It uses the visual division of a plate as a reference to provide the portion recommendation of food groups, such as “*Make half your plate fruits and vegetables*.” When reviewing the photos online, P12 pulled up the MyPlate.gov website and the photo diary side-by-side and described how she visualized the portions in her mind when reviewing food photos. She estimated each of the food groups in the same photo based on the proportion they took in a photo:

“*So that (showing the figure on MyPlate.gov) is what they ideally want you to look for. This little red part is fruits. This is vegetables, this is your grains, this is your protein, and that’s your dairy. In my head, I’m picturing this plate, right? So I’m saying, okay, this person (switching to the photo diary) is a little bit better than average. This (pointing at a region of the photo) is like a lean pork loin. So that’s a lean protein source. Hey, you know, about 30% of their plate is green beans, a vegetable. So that’s good. And then this (pointing at another region of the photo) is their grain, but I can see it’s buttermilk, rich mashed potatoes.*” (P12; In- & out-patient dietitian for 15 years)

P04 similarly reviewed the food photos using the MyPlate method. She categorized the food shown in the photo into food groups and used the MyPlate method to analyze the photo. Similar to P12, she thought the relative portion of the food groups was a good indicator of whether the client ate a balanced diet.

“This *(pointing at a region of the photo) looks like eggs or some sort of breakfast casserole. And then like the cinnamon roll (pointing at another region of the photo), which looks like the biggest piece of food on the plate. If we go by MyPlate, you want a fourth of your plate to be carbohydrates and a fourth protein, and then the other half like vegetables and fruit. So it looks like they can afford to have a bigger portion of protein, which is their eggs. But their carbohydrates just look like the main course of the meal where it should be more of a side.*” (P04; Inpatient dietary intern for 6 months)

P17 also thought that with the photos, she would get a better idea of the distribution of each type of food than with other dietary assessment methods. The visual would also make it easy to teach her clients how to balance their diets.

“*Wow, this would be so much better than a food frequency questionnaire or 24-hour recall. I bet that the portion of food or even what they ate in these photos is probably a lot different than what they would recall. As I was going through it, the first picture [pointing at the first photo], I thought, ‘Wow, this person is going to do really well with balancing foods and variety.’ Then with these photos, we could use a method that we call the plate method, where half of your plate is vegetables and limited portions of grains. I could apply it for really everybody.*” (P17; Outpatient dietitian for 13 years)

Food photos provide affordance for dietitians to assess the variety and balance of food intake from both high-level overviews and within individual meals. These visual features also potentially support dietitians to use photos as examples for dietary education.

### Infer how and where clients ate

Beyond the food content, many participants also paid attention to the eating contexts in photos by identifying where they ate, what social context they were in while eating, and how they prepared their food.

The first type of information participants asked about was the environmental context. All participants tried to identify artifacts in the background to understand the client’s living environment. Objects like a keyboard, the interior of a car, a TV, or a kitchen table, helped participants identify where the clients were having their food. P12 explained what she was looking for in the photos and what questions she would ask a client if these environmental contexts were not obvious from the photos:

"*I would ask them to tell me where they were, something else that would be, are they sitting down at the dinner table and eating, or are they standing up and talking and running around? Are they actually aware of what they’re eating? Where did they eat this? In their car? In the break room?*" (P12; In- & out-patient dietitian for 15 years)

Other than identifying objects from the photos, some participants tried to use food containers or the background in the photos to identify where the clients were when having their meals. For instance, P13 thought that one client may have had a taco in a restaurant because she recognized the taco tray and the plating technique in the photo: “*The plating here*, *there’s a taco holder*, *this is gonna sound funny*, *but there’s a few breweries that have these plates*, *so it just reminds me of a restaurant*. *I like to do a lot of culinary things too*. *So I see culinary techniques used in this dish specifically*. *There’s like a swoop of the Guacamole*, *or the wasabi*, *whatever that they’re using here*. *It looks like a fancier dish*. *There’s dark lighting*, *so they’re probably at a restaurant where people like to sit in the dark*, *they’re not sitting at home*.*”* (P13; private practitioner for 13 years)

She also noticed that across the diary, she could tell there seemed to be quite a few restaurant-like photos. She thought that getting this information was important to provide more actionable recommendations in contexts. For example, she mentioned that she would teach the client how to read a menu and choose their food wisely.

*“So something that I’ve kind of observed is if you’re eating out, you usually eat more in a restaurant setting just cause you’re given more. So being able to see this [eating environment] from the photo, my recommendation there would be, okay, if you’re going to go to a restaurant, look for this on the menu, look for a V. If you’re looking for vegetarian, look for a leaf. Whenever you go to a restaurant, they might have words like fried or other than baked I would coach the client to look for a healthier option.* “(P13; Private practitioner for 8 years)

The second type of contextual information participants looked for was the social context. Participants paid attention to whether clients were eating with other people because that may influence what and how much clients ate. For example, P15 pointed out one photo that may have been taken at a party setting because the client was taking several small pieces of food on a paper plate with disposable utensils.

"*This to me looks like a party. It’s kind of a hodgepodge of different stuff and it’s just like one food item here and there. So you’ve got just a couple of apples, like one cracker with a topping, one little piece of bread with some sauces, some chips, and dip. That tends to be one of those party items. And also it’s on a paper plate. So, it looks like that might be something that they were doing at a party cause all these other food items are on a paper plate and then with the single-use fork*." (P15; Outpatient dietitian for 11 years)

P15 thought that being able to identify social contexts from photos would provide her an opportunity to help her patients navigate their food options in these settings, such as by preparing their own healthy food options or talking through what food they should choose in a party setting.

“*I think a lot of my patients have hard times at parties because there’s a lot of higher fat foods, higher sugar. And looking at these photos, we could try to talk through, what you do. Sometimes we recommend that they actually bring their own food that they know is healthy, that they can kind of contribute to the party. And it’s also good to kind of role-play out before, so they know specific foods that are going to be there, like thinking through what they’re going to choose before they get there so they’re not tempted.*” (P15; Outpatient dietitian for 11 years)

Another type of information participants looked for was food preparation. Participants used kitchenware or ingredient packages as a hint to a client’s willingness or capability of making food at home. P09 noticed food containers that were typically used for food prep and thought that indicated the client’s willingness to prepare their own food: “*The milk containers in the photo mean that they are preparing it at home probably*. *And that means that they’re ready to pack something to go instead of having to buy it*, *which is really good*. *That’s a good indication*. *I like seeing that*” (P09; Outpatient dietitian for 1.5 years). P16 thought the food in the photo was homemade from how it was prepared and presented as well as the cooking equipment shown in the photos.

“*This [pointing to one photo] looks like something that I’ve made at home. I don’t see a restaurant serving a sandwich that looks like that or string beans that look like that. This [pointing to another photo] looks like Kraft macaroni and cheese. They’re using these like batch cooking bowls [pointing to a third photo]. They have some equipment like here’s a colander that they’re using to do these preparations*. *It looks like they’re compiling those ingredients themselves and then kind of pulling those things together into a batch.*” (P16; Private practitioner for 1 year)

Research in patient-provider collaboration in patient-generated data has examined and emphasized the importance of contextual information in data review and reflection (e.g., [3,16,48]). Our findings provide further example and evidence of the type of contextual information dietitians gather from food photos and how they use this information in dietary assessment and recommendation.

### Evaluate the sufficiency of client intake

To understand whether clients had an adequate amount of food intake, participants often tried to find indications of how much food clients actually consumed. Because there were no before-and-after photos provided, participants often thought how much clients consumed was the most difficult information to get from photos. P04 thought having before and after photos could give her a good idea of the actual consumption: “*Maybe taking like before and after pictures*, *like how much they’re being given and how much they actually eat of it*” (P04; Inpatient dietary intern for 6 months). When looking at a photo with a muffin, a tuna package, and peanut butter, P11 wanted to know if participants consumed everything:

“*Did they really eat the whole muffin and did they eat all of the tuna fish package? I can’t tell how many slices of stuff are in there. And I don’t know if they had any peanut butter or peanut butter on the bread. So it doesn’t tell me about what was consumed, how much was even given to them*” (P11; Private practitioner for 35 years).

In addition, participants found it difficult to estimate the portion size from the photos. P05 wished there were easier ways for her to determine portion size and the client’s actual consumption. “*I’m drawn to portion size to kind of estimate how much of a food was consumed*” (P05; Outpatient dietitian for 11 years). Similarly, P3 found it hard to know the actual size of the pizza and the portion of the soup.

“*In some of the photos it’s kinda harder to tell portion size. I don’t know these two slices of pizza; how big they are. Also how much the soup is*” (P03; Inpatient dietary intern for 6 months).

One common strategy that participants in our study mentioned was to suggest clients include a reference object when taking photos. For example, P04 imagined a standardized ruler would help her gauge the portion size:

“*I need some sort of a point of reference to see how big the thing is*. *If there is a way to have a ruler or something*, *just show up on the scale on your photo technology to show me portion sizes*.” (P4; Inpatient dietary intern for 6 months)

Both P13 and P16 suggested that if clients take a few photos from different angles, that will help them identify the depth of the container as well as analyze the amount of food inside.

“*I would say like if it’s like a soup bowl maybe take a picture from the side too, like on top of the side, so we can see how much is in there*.” (P13; Private practitioner for 8 years)

“*If you do a top-down view*, *that’s not very helpful*. *So like I said*, *trying to show a 45-degree angle*. *That way I understand what you’re really having*. *I can understand some depth to that photo that’s being shown*.” (P16; Private practitioner for 1 year)

Estimating portions has been one of the major challenges across dietary assessment methods [4]. Although research shows that image-based dietary record improves portion estimation compared to traditional text-based diaries [10], participants in our study still consider the task challenging. Some systems have started to investigate the use of reference objects in photo-taking [28] and the comparison between before and after photos to better support portion estimation [49]. However, these methods often create an additional burden for users, and therefore the feasibility of these features remains in question [28].

## Discussion

As seen in the study, dietitians adopted a variety of strategies to use visual features to review photo-based diaries, focusing on eating patterns, contexts, variety, and sufficiency. Design of the computer-vision-based food photo diaries can better support individuals and dietitians in data review as well as support individuals to collect and integrate data that facilitate this type of review.

### Design to support analysis over time

Our findings show that when reviewing photo diaries, dietitians in our studies used the visual attributes of photos, such as color or the visual divisions of plates, to identify eating patterns over time, instead of focusing on calorie estimation of individual meals. They looked through and analyzed several photos at a time, sometimes rearranging these photos based on the questions they wanted to answer.

Providing more flexible interaction with the data could potentially facilitate the discovery of eating patterns to support healthy eating and dietary assessment goals. Although many systems already provide different time-period-based views to allow individuals to examine their data, research continues to suggest that more ways to explore data that aligns with individual goals could better facilitate reflection and collaborative review (e.g., [16,17,50,51]). In our study, we observed many participants rearranging photos to answer their review questions, such as the types of food the client ate during meals versus snacks or weekdays versus weekends. Because the food photos in the study were either printed on paper or presented using Google Slides, it was straightforward for participants to freely rearrange the photos. However, in practice, photos presented on client mobile phones or in chronological order on a feed may be difficult for dietitians to create a view to support their analysis. Prior research has explored ways to support expert review by presenting visual summaries based on potential symptom triggers or health goals [16] and using heatmaps to indicate relationships between food and health indicators [18]. Our study provides another example where a better understanding of expert review goals and creating designs with affordance that facilitates these goals have the potential to support better use of tracked data.

Even though dietitian participants in our study wanted to review multiple photos at the same time to identify eating patterns, there is a limitation to how many photos dietitians could reasonably review and analyze at once. There are opportunities for systems to automatically analyze attributes of food photos, such as colors or textures, and provide high-level summaries of eating patterns, such as the proportion of green, red, or yellow vegetables in a meal. These high-level summaries could then serve as a preliminary overview and allow dietitians to develop initial hypotheses for further review. While these summaries may not directly provide caloric or nutrient information, they could potentially support individuals and dietitians to answer questions similar to whether individuals have a balanced diet or enough variety of food throughout a period.

However, computer vision techniques can be limited by the presentation of the food in the photos. For example, lighting in the environment or camera settings could obscure how algorithms identify colors and other attributes of food photos [25,28]. There may be opportunities for human-in-the-loop design to prompt users to augment photos with targeted annotation to support learning in computer vision algorithms. For example, photos taken from the same environment may suggest similar lighting, and that system could better configure the color profile when associating color patterns across photos. Annotation in personal health data is not new and has proven to provide contextual information necessary for making health decisions [3,16,48,51]. Scaffolding the annotation based on the analysis goal (e.g., using color distribution as a proxy to estimate the variety of food eaten) could further support individuals in understanding why and how the data is analyzed. The process of answering these prompts and annotating photos could also help individuals explicitly and intentionally reflect on their data and become more mindful of their eating behaviors and patterns.

### Design to support contextual data analysis

Understanding eating contexts can be critical in dietary assessment. Research shows that individuals tend to eat more when eating in a restaurant setting [52], compared to eating at a dining table at home; Children are more likely to eat fruit and vegetables when they are eating at a table and the TV is off [53]. A better understanding of client lifestyle and constraints also helps health experts to provide emotional support, build relationships, and provide feasible recommendations [2,16]. Prior studies have shown that photos provide contextual information to help health experts understand participant routines [3,16]. In our study, we provide further evidence and examples of what context is important during photo-based dietary assessment and why they are important, such as when dietitians explicitly looked for containers and background objects to help them identify where clients ate and how they prepared their food.

Although photos are potentially more likely to contain contextual information than text-based diaries or food frequency questionnaires, individuals may not always collect this information when taking photos. For example, when taking photos, individuals may only focus on food and not include the containers or background. People who focus on the social benefits of shared food photos may choose to blur the background to protect privacy [54,55] or improve the aesthetic aspects of food photos (e.g., when sharing on Instagram [8]). As prior work suggested, individual needs in different stages of data collection and analysis might not align with health expert goals in review [2,56]. Explicitly surfacing and clarifying these goals as well as communicating potentially conflicting needs could be beneficial to ensure proper data collection. For example, systems could allow users to apply filters to obscure contextual information in social sharing but enable explicit access when working with health experts. Designs could also remind individuals to frame the photo and capture necessary contextual information (e.g., including containers or utensils) when taking photos for healthy eating purposes, as opposed to for social sharing purposes.

Interpreting contextual information often requires dietitian experience and judgment. In our study, we observed dietitians used the information surfaced in photo backgrounds to reason and hypothesize client eating decisions and behavior. They used this contextual information to develop "situated objectivity"—the objective interpretation based on individual situations [57,58]—to the best of their ability in the absence of clients. They also developed other questions they could potentially take to dietary consultation sessions when clients were present.

There are opportunities for system design to support dietitians in context-specific dietary assessment and recommendations. For example, computer vision analysis could support context recognition in food photos. Traditionally, computer vision algorithms often segment photos based on where food might be and focus on identifying what might be inside a plate or a food container. These classifications could result in a mix of outcomes, with some algorithms having better performance in ingredient recognition and others better trained to recognize everyday objects found in the background, such as a computer keyboard (e.g., [25,59]). In practice, as findings from this study show, both types of information (i.e., eating content and context) could be equally important and collectively support the goal of dietary assessment. Based on contextual cues presented in the photos and information available through metadata in photos (e.g., location and time) or other sensors (e.g., social context inferred through microphones [20], systems could then categorize food photos into different frames, building on the concept of “contextual frames [60].” Dietitians could then use these contextual frames to facilitate in-person consultation and provide context-specific recommendations, such as system-supported just-in-time interventions (e.g., [61,62]).

### Future of work for dietary assessment and consultation

With the growing needs and interests in healthy eating and diet, many dietitians today work in remote dietary assessment and consultation settings. These could include synchronous contexts, where dietary reviews are conducted in video conferencing sessions, and asynchronous contexts, where dietitians review dietary intake on an on-demand basis. When more and more clients and patients adopt photo-based food diaries because they are relatively low-burden and socially acceptable, how well these diaries support dietitians in these settings requires further investigation.

Unlike traditional face-to-face or synchronous consultations where dietitians and clients rely on conversations to probe client behavioral contexts, routines, and goals, dietitians working in asynchronous settings may not have the opportunity to develop an understanding of the clients through conversations and long-term working relationships. Photos may provide an opportunity for dietitians to acquire some contextual understandings, such as by inferring the client lifestyle from the background of food photos as we observed in the study. Systems could further involve individual patients or clients in the curation and sharing of visual content and the context of eating. Instead of nudging individuals to collect *more* data, incorporating dietitian review goals could support individuals in collecting *necessary* data, such as by prompting what to include in the food photos and how to take better photos. In particular, while recent research suggests that design supporting eliciting, communicating, and scaffolding individual and health expert goals in the self-tracking process could support better use of the data [56,63], these communications may be overlooked when clients and health experts collaborate asynchronously through technologies. As individual and health expert goals might evolve throughout their healthy eating journey, surfacing and scaffolding these goals, the process to achieve these goals, and the questions they encounter during the process could help health experts better contextualize client data.

One scenario for remote, asynchronous dietary assessments today includes a combination of intelligent system design and remote dietitian consultation (e.g., commercial nutrition services, such as Noom [64], NutritIO [65], and Nourishly [66]). These services often leverage algorithm-based (e.g., image recognition) analysis to provide first-line, real-time support and allow remote, human dietitians to conduct consultation asynchronously or on a less frequent basis. On one hand, these service models allow nutrition experts to support individuals across geographical areas. On the other hand, in many cases, individuals and health experts may need to learn how to work with the algorithm-based analysis to make the best use of this dietary assessment support. Dietitians working on remote dietary assessment and consultation might have different review goals/assumptions (e.g., evaluating balance or variety of eating) from systems designed to support calorie-goal-based analysis (as is often the focus of existing photo-based food AI systems [26]). This discrepancy may create tensions, such as conflicting recommendations, when individuals transition between automatic system support and remote dietitian support. Design to support computer-vision-supported food photo review in these contexts may need further understanding of the workflow, needs, and constraints of this type of interaction. Our research did not specifically work with dietitians or clients who based their food photo review on automatic analysis, but findings from this research could serve as a basis for future investigations into the future of work for dietary assessment and consultation.

## Conclusion

To understand how designs can support expert dietary assessment using photo-based food diaries, we conducted an interview and observation study with 18 dietitians. Our findings show that dietitians used the visual features of food photos to understand the eating patterns over time, the variety of food intake, the contexts of eating, and the sufficiency of food consumption. Based on these findings, we discuss how design could support photo analysis over time and contextual data analysis. By allowing human-in-the-loop annotation based on expert analysis goals, automatic analysis can provide a high-level, personalized summary to support nutrition experts in developing an initial hypothesis for further review. By integrating algorithms that are optimized for identifying background objects and food content, systems could support dietitians to better provide context-specific recommendations. Finally, future research is needed to examine how to better support individuals and dietitians to collaborate with automatic image analysis algorithms.

## Supporting information

S1 File Interview Protocol. (PDF)

S2 File Observation Protocol. (PDF)
