# Correction: Opportunities to design better computer vison-assisted food diaries to support individuals and experts in dietary assessment: An observation and interview study with nutrition experts

**DOI:** 10.1371/journal.pdig.0001097

**Published:** 2025-11-13

**Authors:** Chia-Fang Chung, Pei-Ni Chiang, Connie Ann Tan, Chien-Chun Wu, Haley Schmidt, Aric Kotarski, David Guise

The word “vision-assisted” is misspelled in the article title. The correct title is: Opportunities to design better computer vision-assisted food diaries to support individuals and experts in dietary assessment: An observation and interview study with nutrition experts. The correct citation is: Chung C-F, Chiang P-N, Tan CA, Wu C-C, Schmidt H, Kotarski A, et al. (2024) Opportunities to design better computer vision-assisted food diaries to support individuals and experts in dietary assessment: An observation and interview study with nutrition experts. PLOS Digit Health 3(11): e0000665. https://doi.org/10.1371/journal.pdig.0000665.
