# Supplementary material for: Opportunities to design better computer vison-assisted food diaries to support individuals and experts in dietary assessment: An observation and interview study with nutrition experts
Source: PLOS Digit Health. 2024 Nov 27;3(11):e0000665. doi: 10.1371/journal.pdig.0000665 (PMC11602110; doi:10.1371/journal.pdig.0000665)
Supplement: S1 File — (PDF) [file pdig.0000665.s001.pdf]

1. **How long have you been conducting dietary assessment and consultation professionally?**

2. **What's your current position and title?**

3. **What type of clients or patients do you usually see?**

4. **Can you tell us more about your dietary assessment and consultation experience (both in the current and previous positions, if any)?**

5. **Can you tell us about your experience with photo-based food diaries?**

6. **Let's go back to the food diary you just reviewed. Can you tell me about what was your first impression of this data set?**

    a. **What did you focus on when you first started to review the diary?**

    b. **Why was that the first thing you focused on?**

    c. **How did you do that?**

    d. **(Use the observation note to clarify their strategies and steps for review)**

7. **What did you do in terms of the diary review after the first glance/overview?**
    a. **What were you trying to find?**

    b. **Why was that important?**

    c. **How did you do that?**

    d. **(Use the observation note to clarify their strategies and steps for review)**

8. **What were you looking for in this stage of the diary review?**
    a. **How did you do that?**

    b. **(Use the observation note to clarify their strategies and steps for review)**

9. **What was missing in the diary that you wish you could see from the photos?**

10. **Why was that information important?**

11. **How would you use that information in the dietary consultation for this client?**

12. **If you were able to give this client an instruction for tracking their food using photos, what instructions would you give them? Why?**

13. **[Follow-up questions based on observation notes]**

14. **Is there anything we haven't talked about but you would like to share with us?**
