# Supplementary material for: Opportunities to design better computer vison-assisted food diaries to support individuals and experts in dietary assessment: An observation and interview study with nutrition experts
Source: PLOS Digit Health. 2024 Nov 27;3(11):e0000665. doi: 10.1371/journal.pdig.0000665 (PMC11602110; doi:10.1371/journal.pdig.0000665)
Supplement: S2 File — (PDF) [file pdig.0000665.s002.pdf]

1. **What is the overall process?**

2. **What information are they looking for in each photo?**

3. **what level of detail are they looking for?**

4. **How do they review the photos? Do they use any ways to characterize the photos?**

5. **How do they make assumptions about the information in the food photos (e.g., the food portion or the type of food that is not clear in the photos)?**

6. **If participants annotate in the diary or write notes on another paper, when and how do they do that?**

7. **What questions do participants have about the client and their eating habits based on the photos?**
